# Sex-Specific Association Between the Neutrophil-to-HDL Cholesterol Ratio and Hyperuricemia in Obstructive Sleep Apnea

**DOI:** 10.3390/jcm15103916

**Published:** 2026-05-19

**Authors:** Xiaoyan Fang, Renjiao Tan, Shuhong Fan, Yuanling Mao, Munire Adili, Yubao Wang, Jing Feng

**Affiliations:** Department of Respiratory and Critical Care Medicine, Tianjin Medical University General Hospital, Tianjin 300052, China

**Keywords:** obstructive sleep apnea, hyperuricemia, neutrophil-to-HDL cholesterol ratio (NHR), sex-specific inflammation

## Abstract

**Background:** Obstructive sleep apnea (OSA) has been associated with hyperuricemia, with systemic inflammation as a potential contributing pathway. The neutrophil-to-high-density lipoprotein cholesterol ratio (NHR) reflects both inflammatory and metabolic status, but sex-specific evidence on its association with hyperuricemia in OSA remains limited. This study aimed to investigate the relationship between NHR and hyperuricemia in OSA patients, focusing on sex differences. **Methods:** In this cross-sectional study, 808 OSA patients underwent polysomnography and laboratory assessments. Receiver operating characteristic curves were used to evaluate the diagnostic performance of NHR. Multivariable logistic regression, restricted cubic spline analyses, and subgroup analyses were used to assess the independent association and dose–response relationship between NHR and hyperuricemia. **Results:** Hyperuricemia was present in 396 (49.0%) patients. Among hyperuricemic patients, females displayed significantly milder OSA severity (lower AHI, ODI, and T90%) but higher systemic inflammatory markers, including higher NHRs, compared with males. NHR showed a relatively better diagnostic accuracy for hyperuricemia in females (AUC = 0.683). Higher NHR quartiles were independently associated with hyperuricemia, with stronger effects in females (Q4 vs. Q1: adjusted OR = 2.68, 95% CI: 1.10–6.51, *p* < 0.001). Restricted cubic spline analyses indicated a linear association in females (*p* for nonlinearity = 0.175) but it was nonlinear in males (*p* = 0.010). Subgroup analyses indicated consistent associations in females across age, BMI, AHI, and hyperlipidemia status. **Conclusions:** NHR was associated with hyperuricemia in OSA, with a stronger and linear association in females. As a simple and accessible biomarker, NHR may be valuable for hyperuricemia risk stratification in female OSA patients and may also provide insights into sex-specific inflammatory mechanisms underlying OSA-related metabolic dysfunction.

## 1. Introduction

Obstructive sleep apnea (OSA) is a highly prevalent sleep-related breathing disorder characterized by recurrent upper airway collapse, intermittent hypoxia, and sleep fragmentation [1]. Recent evidence indicates that genetic factors play a critical role in the pathogenesis of OSA [2]. A population-based analysis of OSA prevalence across 16 countries estimated that, worldwide, approximately 936 million adults aged 30–69 years are affected by mild to severe OSA, while about 425 million individuals suffer from moderate to severe OSA [3]. Large epidemiological studies have demonstrated that OSA is strongly associated with metabolic dysfunction, systemic inflammation, and increased cardiovascular morbidity and mortality [4].

Hyperuricemia is a common metabolic abnormality and an established risk factor for cardiovascular and renal diseases [5]. Increasing evidence supports a close association between OSA and hyperuricemia. Emerging evidence has revealed elevated circulating C-reactive protein (CRP), uric acid and glucose levels in hypertensive individuals with comorbid OSA [6]. Intermittent hypoxia promotes purine catabolism and xanthine oxidase activation, leading to excessive uric acid production, while oxidative stress and endothelial dysfunction impair urate excretion [7]. Clinical studies consistently report higher serum uric acid levels and a greater prevalence of hyperuricemia in OSA patients, with uric acid positively correlating with OSA severity and nocturnal hypoxemia [8].

Systemic inflammation represents a critical mechanistic link between OSA and hyperuricemia. OSA-induced hypoxia activates innate immune responses, neutrophil recruitment, and lipid metabolism dysregulation, resulting in a chronic low-grade inflammatory state [9,10,11]. Recently, composite inflammatory indices derived from routine laboratory tests have been proposed as integrative markers of inflammatory–metabolic risk. Among these, the neutrophil-to-high-density lipoprotein cholesterol ratio (NHR) reflects both pro-inflammatory activation and impaired anti-inflammatory lipid function and has shown prognostic value in cardiometabolic disorders [12,13].

Notably, both OSA and hyperuricemia exhibit pronounced sex-specific differences in inflammatory profiles and metabolic vulnerability. Emerging data suggest that women with OSA may experience disproportionately heightened inflammation despite relatively milder respiratory disturbances [14]. Despite growing evidence linking OSA with hyperuricemia and systemic inflammation, several important gaps remain. First, most previous studies have focused on traditional inflammatory markers, while composite indices integrating both inflammatory and metabolic components have been insufficiently explored. NHR, which reflects the balance between pro-inflammatory activity and anti-inflammatory lipid function, has emerged as a promising biomarker in cardiometabolic disorders [15]; however, its role in OSA-related hyperuricemia has not yet been elucidated. Second, sex-stratified analyses in patients with OSA and hyperuricemia are scarce, and the potential heterogeneity in the relationships among inflammatory markers, hyperuricemia, and sex remains poorly understood. Therefore, the present study aimed to investigate the association between NHR and hyperuricemia in patients with OSA, with a particular focus on sex-specific differences and dose–response relationships.

## 2. Methods

### 2.1. Participants and Variables

**Figure 1:**
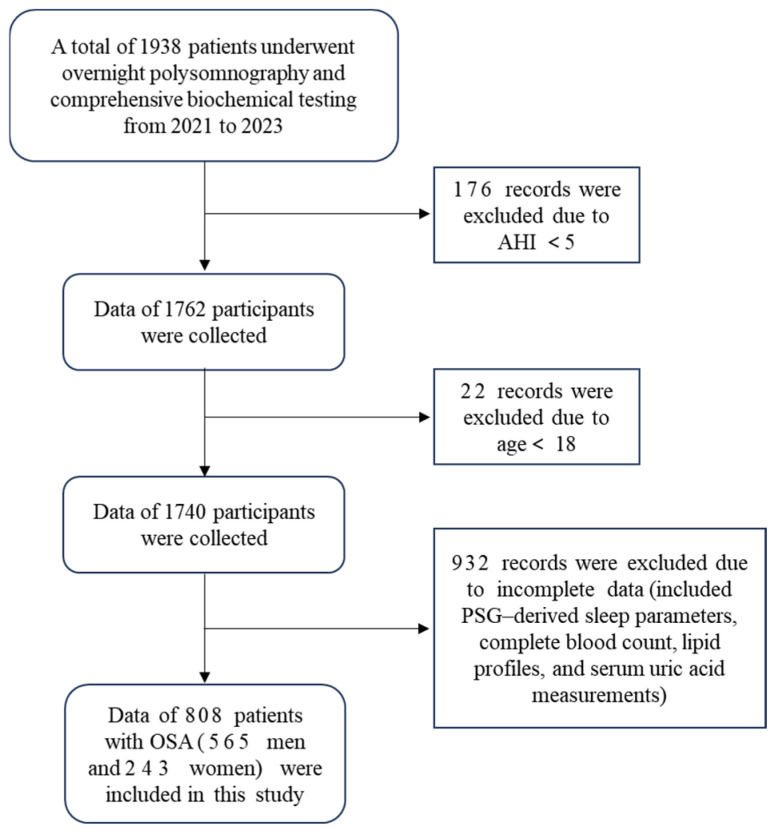
Flow chart of study population selection.

This was a retrospective cross-sectional study; a total of 808 patients diagnosed with OSA were included. Polysomnography (PSG)-derived sleep parameters were collected, along with laboratory measurements including complete blood count, lipid profile, serum uric acid, and blood glucose levels. All variables, including exposure (NHR), outcome (hyperuricemia), and covariates, were predefined prior to analysis. All eligible participants were consecutively recruited from the Sleep Center of Tianjin Medical University General Hospital between January 2021 and January 2023. The exclusion criteria were as follows: age < 18 years; presence of acute illness with an unstable clinical condition; acute infectious disease; current use of medications known to affect serum uric acid levels; use of lipid-lowering agents; and incomplete clinical or laboratory data. The patient selection process is illustrated in Figure 1. All analyses and findings in this study were reported in compliance with the STROBE guidelines, aiming to improve reporting transparency and integrity (Table S1).

### 2.2. Bias and Controlling for Confounding Factors

To reduce selection bias, consecutive patients undergoing PSG were included. Potential confounding factors, including age, body mass index (BMI), OSA severity indices, renal function, and metabolic parameters, were adjusted for in the multivariable regression analyses.

### 2.3. Diagnostic Criteria

OSA was diagnosed when the apnea–hypopnea index (AHI) was ≥5 events per hour. According to AHI values, OSA patients were categorized into three severity groups: mild (5–15 events/h), moderate (>15–30 events/h), and severe (>30 events/h) [16]. Hyperuricemia was assessed based on serum uric acid concentrations. In this study, newly diagnosed hyperuricemia was defined as a serum uric acid level >420 μmol/L (7.0 mg/dL) in men and >360 μmol/L (6.0 mg/dL) in women [17].

### 2.4. Statistical Analysis

Statistical analyses were performed using SPSS software (IBM SPSS Statistics 25, Armonk, NY, USA) and R software (Version 4.4.1). Continuous variables with a normal distribution are presented as means ± standard deviations (SDs), whereas non-normally distributed variables are expressed as medians with interquartile ranges (IQRs). The Shapiro–Wilk test was used to assess data normality.

Comparisons between two groups were conducted using Student’s *t* test for normally distributed variables and the Mann–Whitney *U* test for non-normally distributed variables. Categorical variables were compared using the chi-square test.

Receiver operating characteristic (ROC) curve analysis was applied to evaluate the diagnostic performance of inflammatory markers for identifying hyperuricemia in different OSA populations. Multivariable logistic regression models were constructed to assess the association between NHR and hyperuricemia in patients with OSA. Multivariable logistic regression models were constructed in a stepwise manner. Model 1 was unadjusted; Model 2 was adjusted for key demographic and OSA-related variables (age, BMI, and OSA severity indices); Model 3 was further adjusted for renal function and metabolic parameters. Covariates for the multivariable model were selected according to prior clinical evidence, published literature, and significant variables from the univariate logistic regression, particularly factors affecting uric acid metabolism and systemic inflammation. Multicollinearity among covariates was assessed using the variance inflation factor (VIF); no substantial collinearity was detected, and all VIF values were below 5. Restricted cubic spline (RCS) analyses were performed to examine potential nonlinear relationships between NHR and hyperuricemia. RCS analysis was conducted with predefined knots set at the 5th, 50th, and 95th percentiles of NHR. Subgroup analyses were conducted to evaluate the robustness and consistency of the association between NHR and hyperuricemia across clinically relevant strata. A two-sided *p* value < 0.05 was considered statistically significant. Figures were generated using GraphPad Prism software (version 9.5; GraphPad Software, San Diego, CA, USA).

## 3. Results

### 3.1. Gender Differences in OSA Patients with Normouricemia or Hyperuricemia

A total of 808 patients with OSA were enrolled in this study, and the screening process is presented in Figure 1. Table 1 presents the sex-based differences in baseline characteristics among the patients with OSA, stratified by the presence or absence of hyperuricemia. As shown in the table, among OSA patients with hyperuricemia, female patients exhibited significantly higher waist circumference (WC) and body mass index (BMI) than their male counterparts, along with longer durations of N2 and N3 sleep stages. In contrast, females demonstrated significantly lower AHI, oxygen desaturation index (ODI), and arousal index (ARI), as well as a shorter mean apnea duration. Moreover, female patients had a higher minimum oxygen saturation and a lower proportion of time spent with oxygen saturation below 90% (T90%). Notably, despite the relatively milder nocturnal hypoxemia and sleep-disordered breathing severity, female OSA patients with hyperuricemia exhibited a markedly enhanced systemic inflammatory profile. Specifically, white blood cell counts, neutrophil percentage, absolute neutrophil counts, neutrophil-to-lymphocyte ratio (NLR), lymphocyte-to-monocyte ratio (LMR), systemic immune-inflammation index (SII), and NHR were all significantly higher in females compared with males with hyperuricemia-associated OSA (all *p* < 0.05).

### 3.2. Within-Gender Comparisons Between OSA Patients with Normouricemia and Hyperuricemia

We further examined sex-specific differences in clinical characteristics between OSA patients with and without hyperuricemia. The results demonstrated that, in males, females, and in the overall cohort, patients with OSA and concomitant hyperuricemia exhibited significantly higher waist circumference, BMI, AHI, and ODI compared with those with normouricemia. In addition, the hyperuricemia group showed a significantly lower minimum oxygen saturation (lowest SaO_2_) and a higher proportion of time spent with oxygen saturation below 90%. Compared with normouricemic OSA patients, those with hyperuricemia displayed markedly elevated systemic inflammatory markers, including WBC, neutrophil count (Neu), SII, SIRI, MHR, and NHR (Table 2). Notably, the magnitude of these inflammatory differences was more pronounced in female patients, suggesting a stronger association between hyperuricemia and systemic inflammation in women with OSA.

### 3.3. NHR as an Optimal Inflammatory Marker for Identifying Hyperuricemia in Female OSA Patients

**Figure 2:**
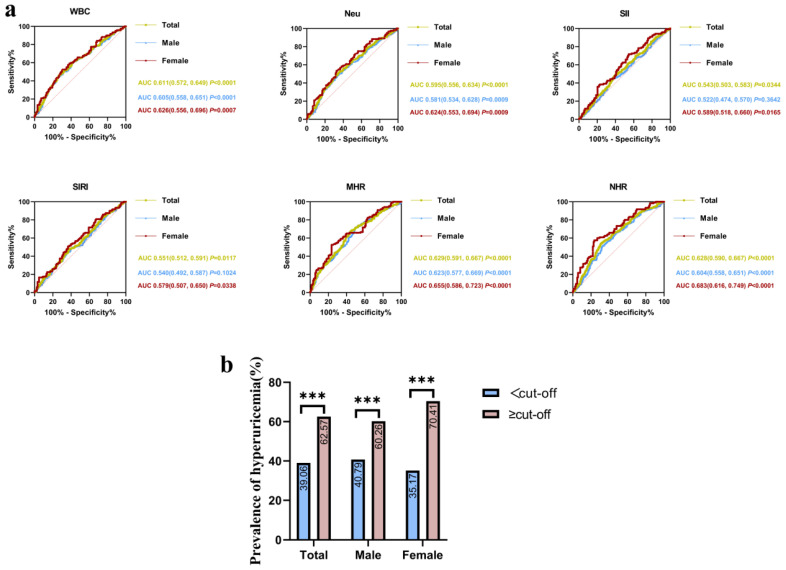
(**a**) ROC curve analysis of inflammatory markers for identifying hyperuricemia in subgroups of patients with OSA. (**b**) Differences in the prevalence of hyperuricemia between the lower and higher NHR groups among different subgroups of the OSA population (*** *p* < 0.001). Abbreviations: WBC, white blood cell count; Neu, neutrophil count; SII, systemic immune-inflammation index; SIRI, systemic inflammation response index; MHR, monocyte-to-HDL ratio; NHR, neutrophil-to-HDL ratio.

ROC curve analyses were performed to evaluate the discriminatory performance of inflammatory markers for identifying hyperuricemia among OSA patients in the overall cohort and in the sex-stratified subgroups (Figure 2a). The results indicated that NHR exhibited the strongest diagnostic performance for hyperuricemia in OSA patients. The AUCs for NHR were 0.628 (95% CI: 0.590–0.667, *p* < 0.0001), 0.604 (95% CI: 0.558–0.651, *p* < 0.0001) and 0.683 (95% CI: 0.616–0.749, *p* < 0.0001) for all patients, male patients, and female patients, respectively. Notably, NHR demonstrated relatively better discriminative ability in female OSA patients compared with males. In female OSA patients, the optimal cutoff value of NHR for identifying hyperuricemia was 3.76, yielding a sensitivity of 57.5% and a specificity of 76.4%. Based on the optimal NHR cutoff values established for different populations, OSA patients were stratified accordingly. The results suggested that patients with NHR values above the cutoff exhibited a significantly higher prevalence of hyperuricemia compared with those below the cutoff, with a robust statistical significance (*p* < 0.001) (Figure 2b). Notably, this association was most pronounced among female patients.

### 3.4. Elevated NHR Is Independently Associated with Hyperuricemia in OSA, with a Stronger Association in Females

Since the ROC analyses indicated that NHR is a robust marker for hyperuricemia—particularly in female OSA patients—we further explored the independent association between NHR and hyperuricemia using multivariable logistic regression models stratified by sex (Table 3). NHR was categorized into quartiles (Q1–Q4), with the lowest quartile serving as the reference.

In male OSA patients, higher NHR levels were significantly associated with increased odds of hyperuricemia. In the crude model (Model 1), the odds ratios (ORs) for hyperuricemia progressively increased across NHR quartiles, reaching 2.80 (95% CI: 1.73–4.54) in Q4 (*p* < 0.001). Uric acid levels are known to be associated with renal function, BMI and age; therefore, these factors were adjusted for as confounders in the present study. After adjustment for age, BMI, and OSA-related severity indices (Model 2), this association remained statistically significant for Q3 and Q4. Further adjustment for renal function and metabolic parameters (Model 3) slightly attenuated the effect sizes but did not abolish the association, with Q3 and Q4 remaining independently associated with hyperuricemia. Notably, the association between elevated NHR and hyperuricemia was substantially stronger in female OSA patients. In the crude model, women in the highest NHR quartile exhibited a nearly fivefold increased odds of hyperuricemia compared with those in Q1 (OR = 4.89, 95% CI: 2.27–10.52; *p* < 0.001). Although adjustment for confounding variables attenuated the magnitude of the association, NHR remained an independent indicator of hyperuricemia in females even in the fully adjusted model (Model 3), with an OR of 2.68 (95% CI: 1.10–6.51; *p* = 0.029) for Q4.

Collectively, these findings indicate a dose–response relationship between increasing NHR levels and the risk of hyperuricemia in OSA patients, independent of obesity, OSA severity, renal function, and metabolic status. Importantly, the association is more pronounced and robust in female patients, further suggesting that NHR could serve as a particularly sensitive inflammatory indicator for identifying hyperuricemia in women with OSA.

### 3.5. Linear Dose–Response Association Between NHR and Hyperuricemia in Female OSA Patients

**Figure 3:**
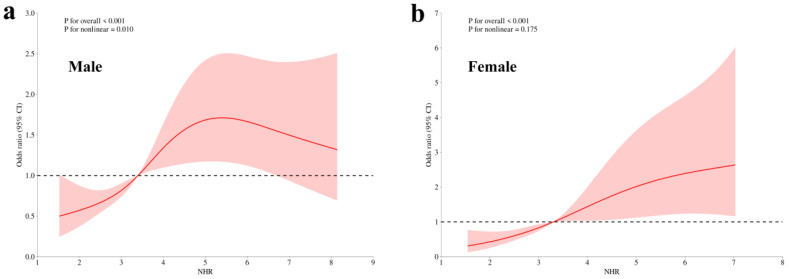
RCS analysis of the association between NHR and the risk of hyperuricemia. (**a**) Nonlinear association between NHR and hyperuricemia risk in males; (**b**) Linear association between NHR and hyperuricemia risk in females.

RCS analyses were conducted to examine the dose–response relationship between NHR and hyperuricemia in OSA patients, stratified by sex (Figure 3). In both males and females, higher NHR levels were significantly associated with an increased risk of hyperuricemia (*p* for overall <0.001). Notably, while a significant nonlinear association was observed in males, the association in females followed an approximately linear pattern, with no evidence of nonlinearity. This finding indicates a more stable and consistent dose–response relationship between NHR and hyperuricemia in female OSA patients, further supporting the stronger and more robust association observed in women.

### 3.6. Consistent Association Between NHR and Hyperuricemia Across Clinical Subgroups in Female OSA Patients

**Figure 4:**
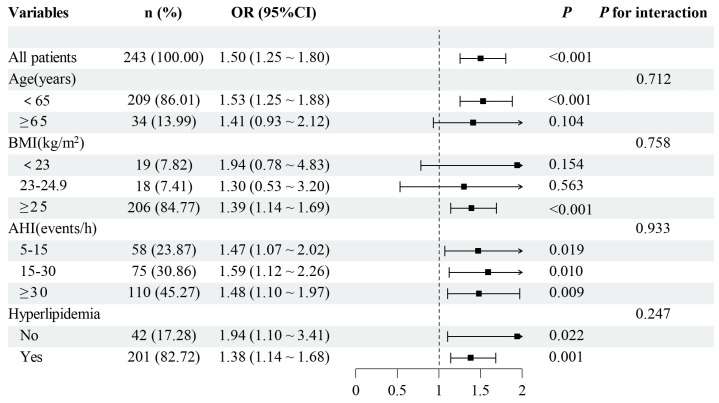
Subgroup analysis of the association between NHR and hyperuricemia in female OSA patients. Abbreviations: BMI, body mass index; AHI, apnea-hypopnea index.

Given the stronger association observed in women, we performed subgroup analyses restricted to female OSA patients to assess the robustness of the association between NHR and hyperuricemia (Figure 4). In the overall female cohort, elevated NHR was significantly associated with an increased risk of hyperuricemia (OR = 1.50, 95% CI: 1.25–1.80; *p* < 0.001). Stratified analyses demonstrated that this positive association was generally consistent across clinically relevant subgroups, including those based on age, BMI, OSA severity (AHI), and hyperlipidemia status.

Specifically, the association remained significant in women younger than 65 years and in those with a BMI ≥ 25 kg/m^2^, while significant associations were observed across all AHI categories. Moreover, elevated NHR was associated with hyperuricemia regardless of hyperlipidemia status. Importantly, none of the interaction tests reached statistical significance, indicating that the association between NHR and hyperuricemia in female OSA patients was not materially modified by these clinical characteristics, underscoring the stability and robustness of this relationship in women.

## 4. Discussion

In this retrospective cross-sectional study, we systematically investigated the association between OSA and hyperuricemia in a cohort of patients. Our findings suggest that inflammatory indices—particularly NHR—are independently associated with hyperuricemia among OSA patients. These results suggest a potential interplay between intermittent hypoxia, systemic inflammation, lipid metabolism dysregulation, and uric acid metabolism in OSA.

In the present study, the prevalence of hyperuricemia was 48.8% among male patients with OSA and 49.4% among female patients with OSA. In a large weighted sample representing 25,354,276 individuals from the KNHANES, 12.2% had a high OSA risk. The high OSA risk group had higher mean serum uric acid levels (5.9 mg/dL) and a higher prevalence of gout (6.6%) than the intermediate- and low-risk groups, and high OSA risk was associated with hyperuricemia after adjustment (adjusted OR: 1.462) [18]. Elevated serum uric acid levels are frequently observed in OSA patients, and OSA severity positively correlates with uric acid levels, which may contribute to increased cardiovascular risk and mortality in this population [19]. Additionally, the evidence indicates that patients with severe OSA have significantly higher serum uric acid levels than both control individuals and those with milder OSA [5]. Uric acid is generated through the oxidative degradation of hypoxanthine and xanthine, which is catalyzed by xanthine oxidoreductase (XOR), which exists in two interconvertible forms: xanthine dehydrogenase (XDH) and xanthine oxidase (XO) [20]. Under physiological conditions, XOR expression is generally low; however, hypoxia markedly upregulates its transcription. In parallel, hypoxic conditions accelerate the conformational conversion of XDH to XO, thereby promoting increased uric acid production [21].

Since the pro-inflammatory effects of neutrophils can synergize with the anti-inflammatory effects of HDL, NHR has been proposed as a promising inflammatory biomarker [22,23]. NHR has been recognized as an inflammatory biomarker in a variety of diseases. Previous studies have demonstrated that NHR is significantly and positively correlated with the AHI and ODI in patients with OSA, and has been associated with increased cardiovascular risk in this population [24]. As an HDL-based inflammatory index, NHR also reflects the underlying metabolic homeostasis status. Intermittent hypoxia (IH) and systemic inflammation in OSA lead to renal endothelial dysfunction and microvascular damage, thereby reducing the renal clearance of uric acid. Therefore, the observed association between NHR and hyperuricemia may partly reflect underlying renal dysfunction, and NHR could serve as a potential mediator of OSA-related renal injury. In this study, renal function was only assessed based on serum creatinine, without employing more sensitive indicators such as the estimated glomerular filtration rate (eGFR) or chronic kidney disease staging. Further studies are required to optimize the renal function evaluation system in the future.

OSA in women is frequently underdiagnosed and often presents with atypical symptoms, which may lead to an underestimation of disease burden when assessed using conventional indices such as the AHI [25,26]. Beyond clinical presentation, sex-related biological differences are being increasingly recognized, including variations in endothelial dysfunction, oxidative stress responses, and inflammatory regulation, all of which may contribute to differential cardiometabolic risk profiles in women with OSA [25]. Hormonal factors, particularly the decline in estrogen after menopause, may further exacerbate these alterations and increase susceptibility to metabolic dysfunction. The stronger association observed between NHR and hyperuricemia in female patients with OSA may have several plausible biological explanations. First, sex hormones are known to play an important role in uric acid metabolism and inflammatory regulation. Clinical studies have reported that estradiol levels are significantly reduced in patients with hyperuricemia [27]. Accumulating evidence further suggests that estrogen may suppress uric acid production in both the intestine and systemic circulation by downregulating xanthine oxidase activity [28]. In addition, estrogen levels are closely related to HDL-C concentrations. Previous studies have indicated that estrogen may upregulate the expression of lecithin–cholesterol acyltransferase (LCAT), thereby promoting HDL-C synthesis and reverse cholesterol transport [29]. In addition, experimental evidence supports the presence of sex-dependent inflammatory responses to IH. In a murine model of chronic intermittent hypoxia, female mice exposed to mild hypoxic stress exhibited higher levels of pro-inflammatory cytokines (e.g., IL-6 and TNF-α), lower anti-inflammatory IL-10 levels, and an increased IL-6/IL-10 ratio compared with males [30]. This interplay between hormonal regulation, lipid metabolism, and inflammation may partly explain the relatively stronger association between NHR and hyperuricemia observed in female patients. These findings suggest that biological responses to IH may differ by sex and provide a potential mechanistic framework for the observed sex-specific associations in our study. However, whether such experimental findings fully translate to clinical populations requires further investigation.

Chronic intermittent hypoxia in OSA activates inflammatory signaling pathways, including TLR4-related responses, and contributes to oxidative stress and lipid metabolism dysregulation [31]. Chronic inflammation is widely recognized as an independent risk factor for hyperuricemia and gout [32]. Moreover, accumulating evidence indicates that uric acid can activate the TLR4-NLRP3 inflammasome, thereby triggering inflammatory signaling pathways [33]. When uric acid levels are elevated, receptor for advanced glycation end products (RAGE) signaling is stimulated, leading to activation of nuclear factor-κB (NF-κB) and subsequent transcription and release of intracellular pro-inflammatory cytokines [34]. Therefore, a bidirectional interaction between hyperuricemia and inflammation has been proposed. As a composite inflammatory biomarker, the relationship between NHR and hyperuricemia remains poorly explored, and, to date, no studies have specifically examined this association in patients with OSA.

From a clinical perspective, NHR represents a simple, inexpensive, and readily available biomarker derived from routine blood tests, making it highly applicable in real-world practice. The pronounced association between NHR and hyperuricemia in female OSA patients highlights the potential importance of sex-specific risk stratification in this population. Elevated NHR may help identify patients at higher risk; however, its clinical utility requires validation in prospective studies. While early identification of individuals with elevated NHR could improve metabolic monitoring and personalized management, its impact on clinical outcomes remains to be determined. Several limitations of the present study should be acknowledged. First, this was a single-center, cross-sectional study, which precludes causal inference between NHR and hyperuricemia in patients with OSA. Specifically, it cannot be determined whether elevated NHR precedes the development of hyperuricemia or whether hyperuricemia itself contributes to increased inflammatory marker levels. Additionally, reverse causality cannot be excluded, as uric acid has been shown to participate in inflammatory signaling and may influence systemic inflammatory status. Both elevated NHR and hyperuricemia may reflect downstream manifestations of shared upstream mechanisms, such as IH, adiposity, or broader metabolic dysregulation associated with OSA. Second, the sample size was relatively limited, particularly in the sex-stratified and subgroup analyses, which may have reduced the statistical power to detect weaker associations or interaction effects. In addition, the high exclusion rate may compromise the representativeness of the study population and restrict the generalizability of our findings. Third, residual confounding cannot be excluded. As a retrospective study, detailed data on diet, alcohol intake, physical activity, and medication use were unavailable, and renal function was only assessed based on serum creatinine. Finally, menopausal status was not recorded; given the role of estrogen in HDL metabolism and uric acid homeostasis, this may have influenced the observed sex-specific associations. Future multicenter, prospective studies with larger sample sizes are warranted to validate our findings and clarify underlying mechanisms.

## 5. Conclusions

In conclusion, this study suggests that hyperuricemia is highly prevalent among patients with obstructive sleep apnea. We found that NHR is an independent inflammatory marker associated with hyperuricemia in OSA, with consistently stronger associations observed in female patients. NHR showed superior discriminatory performance for hyperuricemia, particularly in women, and exhibited a dose–response relationship across multiple analytical models. Collectively, our findings provide preliminary evidence indicating that inflammatory and metabolic markers should be integrated into OSA risk assessment, and offer a basis for future mechanistic and interventional studies.

## Figures and Tables

**Table 1:** Gender differences in OSA patients with normouricemia or hyperuricemia.

	Normouricemia			Hyperuricemia		
Variables	Male (n = 289)	Female (n = 123)	*p*	Male (n = 276)	Female (n = 120)	*p*
Age, years	46.00 (38.00, 58.00)	52.00 (37.00, 62.00)	0.294	38.50 (33.00, 49.00)	36.00 (28.00, 48.00)	0.047
NC, cm	42.00 (40.00, 44.00)	38.00 (35.00, 40.50)	<0.001	42.00 (41.00, 45.00)	41.00 (39.00, 42.25)	<0.001
WC, cm	103.00 (95.00, 110.00)	101.00 (90.00, 111.00)	0.158	107.00 (99.00, 118.00)	111.50 (100.00, 122.50)	0.022
ESS, score	8.00 (4.00, 13.00)	5.00 (3.00, 9.00)	<0.001	8.00 (4.00, 13.25)	4.00 (2.00, 9.00)	<0.001
BMI, kg/m^2^	28.06 (25.62, 31.14)	30.11 (24.98, 35.04)	0.026	30.40 (27.62, 34.58)	36.19 (31.86, 42.34)	<0.001
AHI, /h	50.40 (26.10, 69.40)	24.80 (13.95, 48.60)	<0.001	60.95 (32.25, 77.40)	29.30 (15.90, 69.12)	<0.001
ODI, /h	42.90 (18.00, 63.90)	19.80 (11.45, 40.10)	<0.001	53.50 (25.45, 74.12)	29.10 (11.62, 58.55)	<0.001
ARI, /h	30.80 (16.40, 45.20)	17.00 (10.45, 26.85)	<0.001	30.15 (16.98, 50.50)	16.95 (10.67, 31.33)	<0.001
REM duration, s	47.50 (29.00, 64.40)	52.00 (29.25, 71.00)	0.324	44.15 (29.00, 63.85)	47.25 (24.50, 68.62)	0.989
N1 duration, s	122.50 (68.50, 182.00)	69.50 (46.00, 100.00)	<0.001	117.05 (79.72, 196.05)	77.75 (48.38, 112.88)	<0.001
N2 duration, s	170.50 (125.00, 223.50)	199.60 (147.50, 242.25)	0.002	167.25 (112.75, 215.07)	175.05 (136.88, 227.43)	0.110
N3 duration, s	15.50 (0.50, 38.50)	31.50 (11.25, 61.50)	<0.001	15.00 (0.00, 40.12)	42.00 (17.38, 67.88)	<0.001
Average apnea duration, s	22.90 (18.00, 28.80)	15.70 (13.00, 19.05)	<0.001	22.15 (16.40, 27.22)	13.65 (11.78, 17.00)	<0.001
REM AHI, /h	48.90 (24.00, 62.10)	37.70 (16.30, 54.40)	0.013	56.80 (36.55, 70.92)	39.40 (17.30, 73.95)	0.048
NREM AHI, /h	49.80 (24.50, 69.40)	22.30 (10.85, 47.30)	<0.001	60.55 (30.10, 77.75)	26.65 (12.78, 67.62)	<0.001
AverageSaO_2_, %	94.00 (92.00, 95.00)	95.00 (93.00, 96.00)	<0.001	93.00 (90.00, 95.00)	94.00 (92.00, 96.00)	<0.001
LowestSaO_2_, %	77.00 (67.00, 85.00)	83.00 (77.00, 88.00)	<0.001	72.00 (60.75, 81.00)	80.50 (70.75, 86.25)	<0.001
T90, %	7.00 (0.40, 23.30)	0.60 (0.01, 6.90)	<0.001	14.10 (2.18, 34.90)	1.40 (0.10, 12.85)	<0.001
WBC, 10 × 9/L	6.40 (5.50, 7.48)	6.38 (5.17, 7.72)	0.812	7.18 (5.97, 8.25)	7.32 (5.82, 8.80)	0.319
RBC, 10 × 12/L	5.00 (4.67, 5.28)	4.42 (4.16, 4.68)	<0.001	5.12 (4.80, 5.38)	4.62 (4.32, 4.84)	<0.001
HGB, g/L	151.00 (143.00, 159.00)	129.00 (123.00, 136.50)	<0.001	153.00 (145.00, 160.00)	132.00 (124.75, 138.00)	<0.001
PLT, 10 × 9/L	231.00 (200.00, 263.00)	252.00 (214.00, 308.00)	<0.001	239.00 (210.75, 277.00)	278.00 (231.75, 322.50)	<0.001
NEU%	55.80 (50.30, 61.50)	54.70 (49.60, 62.25)	0.663	56.30 (51.18, 61.23)	58.35 (52.58, 62.92)	0.041
LYMPH%	32.30 (27.00, 37.80)	35.00 (28.80, 38.90)	0.054	32.70 (27.87, 37.90)	32.70 (27.28, 38.23)	0.519
MON%	7.70 (6.70, 9.00)	7.20 (6.10, 7.95)	<0.001	7.90 (6.80, 8.90)	6.85 (5.77, 7.60)	<0.001
EOS%	2.40 (1.50, 3.60)	1.60 (1.20, 2.60)	<0.001	2.20 (1.60, 3.10)	1.75 (1.17, 2.60)	<0.001
BAS%	0.60 (0.40, 0.80)	0.50 (0.40, 0.70)	0.061	0.60 (0.40, 0.70)	0.40 (0.30, 0.50)	<0.001
NEU#, 10 × 9/L	3.44 (2.94, 4.40)	3.58 (2.69, 4.55)	0.983	3.88 (3.13, 4.93)	4.29 (3.33, 5.27)	0.067
LYMPH#, 10 × 9/L	2.07 (1.64, 2.52)	2.17 (1.72, 2.61)	0.165	2.24 (1.84, 2.70)	2.26 (1.90, 2.75)	0.677
MON#, 10 × 9/L	0.50 (0.42, 0.59)	0.45 (0.37, 0.53)	<0.001	0.56 (0.45, 0.66)	0.49 (0.40, 0.60)	<0.001
EOS#, 10 × 9/L	0.14 (0.09, 0.24)	0.11 (0.07, 0.17)	<0.001	0.16 (0.11, 0.23)	0.13 (0.09, 0.21)	0.005
BAS#, 10 × 9/L	0.04 (0.02, 0.05)	0.03 (0.02, 0.04)	0.047	0.04 (0.03, 0.05)	0.03 (0.02, 0.04)	<0.001
NLR	1.73 (1.31, 2.27)	1.56 (1.26, 2.13)	0.154	1.72 (1.35, 2.17)	1.79 (1.37, 2.27)	0.311
LMR	4.12 (3.28, 5.03)	4.92 (3.89, 5.68)	<0.001	4.22 (3.31, 5.30)	4.93 (3.71, 5.91)	<0.001
SII	398.55 (286.10, 537.11)	418.44 (298.80, 581.72)	0.302	406.13 (292.26, 557.59)	456.44 (364.30, 673.78)	<0.001
SIRI	0.89 (0.60, 1.21)	0.75 (0.52, 1.14)	0.015	0.95 (0.64, 1.32)	0.89 (0.60, 1.25)	0.312
MHR	0.44 (0.35, 0.59)	0.38 (0.28, 0.46)	<0.001	0.52 (0.42, 0.67)	0.46 (0.34, 0.57)	<0.001
NHR	3.17 (2.44, 4.15)	2.84 (2.05, 3.75)	0.009	3.71 (2.89, 4.80)	4.03 (2.73, 5.06)	0.486
TC, mmol/L	4.69 (4.10, 5.41)	4.94 (4.33, 5.62)	0.039	4.92 (4.37, 5.59)	4.93 (4.40, 5.51)	0.784
TG, mmol/L	1.57 (1.18, 2.35)	1.45 (1.06, 2.05)	0.035	2.12 (1.55, 3.06)	1.85 (1.55, 2.49)	0.030
HDL-C, mmol/L	1.10 (0.98, 1.24)	1.25 (1.04, 1.43)	<0.001	1.05 (0.94, 1.16)	1.09 (0.97, 1.23)	0.022
LDL-C, mmol/L	2.91 (2.30, 3.55)	3.07 (2.47, 3.41)	0.616	3.05 (2.60, 3.51)	3.15 (2.61, 3.68)	0.492
Serum creatinine, umol/L	63.00 (56.00, 71.00)	50.00 (42.50, 56.00)	<0.001	68.00 (61.00, 78.25)	49.00 (41.75, 54.00)	<0.001
Glucose, mmol/L	5.00 (4.50, 6.00)	5.00 (4.60, 5.90)	0.705	4.90 (4.50, 5.62)	5.40 (4.80, 6.55)	<0.001
Hyperlipidemia, n (%)			0.099			0.041
No	94 (32.53)	30 (24.39)		50 (18.12)	12 (10.00)	
Yes	195 (67.47)	93 (75.61)		226 (81.88)	108 (90.00)	
Diabetes, n (%)			0.811			0.001
No	247 (85.47)	104 (84.55)		254 (92.03)	97 (80.83)	
Yes	42 (14.53)	19 (15.45)		22 (7.97)	23 (19.17)	

NC, neck circumference; WC, waist circumference; ESS, Epworth Sleepiness Scale; BMI, body mass index; AHI, apnea-hypopnea index; ODI, oxygen desaturation index; ARI, arousal index; REM duration, rapid eye movement sleep duration; N1 duration, stage N1 sleep duration; N2 duration, stage N2 sleep duration; N3 duration, stage N3 sleep duration; REM AHI, apnea-hypopnea index in REM sleep; NREM AHI, apnea-hypopnea index in NREM sleep; AverageSaO_2_, mean percutaneous oxygen saturation; LowestSaO_2_, minimum percutaneous oxygen saturation; T90, percentage of total sleep time with SpO_2_ < 90%; WBC, white blood cell count; RBC, red blood cell count; HGB, hemoglobin level; PLT, platelet count; NEU%, neutrophil percentage; LYMPH%, lymphocyte percentage; MON%, monocyte percentage; EOS%, eosinophil percentage; BAS%, basophil percentage; NEU#, neutrophil count; LYMPH#, lymphocyte count; MON#, monocyte count; EOS#, eosinophil count; BAS#, basophil count; NLR, neutrophil-to-lymphocyte ratio; LMR, lymphocyte-to-monocyte ratio; SII, systemic immune-inflammation index; SIRI, systemic inflammation response index; MHR, monocyte-to-HDL ratio; NHR, neutrophil-to-HDL ratio; TC, total cholesterol; TG, triglycerides; HDL-C, high-density lipoprotein cholesterol; LDL-C, low-density lipoprotein cholesterol.

**Table 2:** Gender-stratified comparison of normouricemia and hyperuricemia in OSA patients.

	OSA			Male			Female		
Variables	Normouricemia (n = 412)	Hyperuricemia (n = 396)	*p*	Normouricemia (n = 289)	Hyperuricemia (n = 276)	*p*	Normouricemia (n = 123)	Hyperuricemia (n = 120)	*p*
Age, years	47.50 (37.00, 58.00)	38.00 (31.00, 49.00)	<0.001	46.00 (38.00, 58.00)	38.50 (33.00, 49.00)	<0.001	52.00 (37.00, 62.00)	36.00 (28.00, 48.00)	<0.001
Nc, cm	41.00 (38.00, 43.12)	42.00 (40.00, 45.00)	<0.001	42.00 (40.00, 44.00)	42.00 (41.00, 45.00)	0.002	38.00 (35.00, 40.50)	41.00 (39.00, 42.25)	<0.001
Wc, cm	102.00 (94.00, 111.00)	109.00 (100.00, 120.00)	<0.001	103.00 (95.00, 110.00)	107.00 (99.00, 118.00)	<0.001	101.00 (90.00, 111.00)	111.50 (100.00, 122.50)	<0.001
ESS, score	7.00 (4.00, 12.00)	6.00 (4.00, 12.00)	0.453	8.00 (4.00, 13.00)	8.00 (4.00, 13.25)	0.666	5.00 (3.00, 9.00)	4.00 (2.00, 9.00)	0.423
BMI, kg/m^2^	28.31 (25.51, 32.28)	31.83 (28.29, 37.39)	<0.001	28.06 (25.62, 31.14)	30.40 (27.62, 34.58)	<0.001	30.11 (24.98, 35.04)	36.19 (31.86, 42.34)	<0.001
AHI, /hr	41.80 (20.32, 67.05)	51.35 (25.25, 75.73)	<0.001	50.40 (26.10, 69.40)	60.95 (32.25, 77.40)	<0.001	24.80 (13.95, 48.60)	29.30 (15.90, 69.12)	0.168
ODI, /hr	35.30 (15.35, 59.87)	44.60 (21.75, 70.32)	<0.001	42.90 (18.00, 63.90)	53.50 (25.45, 74.12)	<0.001	19.80 (11.45, 40.10)	29.10 (11.62, 58.55)	0.052
ARI, /hr	24.25 (13.28, 41.82)	24.45 (14.28, 46.55)	0.382	30.80 (16.40, 45.20)	30.15 (16.98, 50.50)	0.505	17.00 (10.45, 26.85)	16.95 (10.67, 31.33)	0.582
REM duration, s	49.25 (29.00, 65.50)	44.50 (27.38, 64.12)	0.158	47.50 (29.00, 64.40)	44.15 (29.00, 63.85)	0.365	52.00 (29.25, 71.00)	47.25 (24.50, 68.62)	0.234
N1 duration, s	99.75 (61.00, 161.90)	101.75 (63.00, 171.03)	0.303	122.50 (68.50, 182.00)	117.05 (79.72, 196.05)	0.392	69.50 (46.00, 100.00)	77.75 (48.38, 112.88)	0.268
N2 duration, s	179.75 (131.00, 229.00)	170.75 (121.00, 219.12)	0.045	170.50 (125.00, 223.50)	167.25 (112.75, 215.07)	0.254	199.60 (147.50, 242.25)	175.05 (136.88, 227.43)	0.029
N3 duration, s	21.25 (1.50, 47.12)	21.75 (1.00, 51.50)	0.487	15.50 (0.50, 38.50)	15.00 (0.00, 40.12)	0.894	31.50 (11.25, 61.50)	42.00 (17.38, 67.88)	0.194
Average apnea duration, s	20.20 (15.20, 26.00)	18.95 (14.00, 25.22)	0.031	22.90 (18.00, 28.80)	22.15 (16.40, 27.22)	0.214	15.70 (13.00, 19.05)	13.65 (11.78, 17.00)	0.003
REM AHI, /h	45.10 (21.48, 60.00)	54.70 (28.67, 71.38)	<0.001	48.90 (24.00, 62.10)	56.80 (36.55, 70.92)	<0.001	37.70 (16.30, 54.40)	39.40 (17.30, 73.95)	0.180
NREM AHI, /h	40.70 (18.48, 67.35)	51.35 (20.82, 75.60)	0.002	49.80 (24.50, 69.40)	60.55 (30.10, 77.75)	0.002	22.30 (10.85, 47.30)	26.65 (12.78, 67.62)	0.207
AverageSaO_2_, %	94.00 (92.00, 96.00)	93.00 (91.00, 95.00)	<0.001	94.00 (92.00, 95.00)	93.00 (90.00, 95.00)	<0.001	95.00 (93.00, 96.00)	94.00 (92.00, 96.00)	0.066
LowestSaO_2_, %	80.00 (69.00, 86.00)	74.00 (63.00, 83.00)	<0.001	77.00 (67.00, 85.00)	72.00 (60.75, 81.00)	<0.001	83.00 (77.00, 88.00)	80.50 (70.75, 86.25)	0.040
T90, %	4.10 (0.16, 21.20)	9.60 (0.80, 28.06)	<0.001	7.00 (0.40, 23.30)	14.10 (2.18, 34.90)	<0.001	0.60 (0.01, 6.90)	1.40 (0.10, 12.85)	0.228
WBC, 10 × 9/L	6.39 (5.38, 7.53)	7.20 (5.92, 8.39)	<0.001	6.40 (5.50, 7.48)	7.18 (5.97, 8.25)	<0.001	6.38 (5.17, 7.72)	7.32 (5.82, 8.80)	<0.001
RBC, 10×12/L	4.80 (4.44, 5.19)	4.93 (4.62, 5.29)	<0.001	5.00 (4.67, 5.28)	5.12 (4.80, 5.38)	0.003	4.42 (4.16, 4.68)	4.62 (4.32, 4.84)	<0.001
HGB, g/L	145.00 (133.00, 156.00)	148.00 (135.00, 157.00)	0.106	151.00 (143.00, 159.00)	153.00 (145.00, 160.00)	0.129	129.00 (123.00, 136.50)	132.00 (124.75, 138.00)	0.132
PLT, 10×9/L	235.00 (203.75, 275.25)	248.00 (214.75, 291.00)	0.002	231.00 (200.00, 263.00)	239.00 (210.75, 277.00)	0.030	252.00 (214.00, 308.00)	278.00 (231.75, 322.50)	0.026
NEU%	55.25 (50.05, 61.82)	56.55 (51.27, 61.92)	0.307	55.80 (50.30, 61.50)	56.30 (51.18, 61.23)	0.927	54.70 (49.60, 62.25)	58.35 (52.58, 62.92)	0.053
LYMPH%	33.15 (27.50, 38.52)	32.70 (27.60, 37.92)	0.521	32.30 (27.00, 37.80)	32.70 (27.87, 37.90)	0.660	35.00 (28.80, 38.90)	32.70 (27.28, 38.23)	0.069
MON%	7.60 (6.40, 8.70)	7.55 (6.50, 8.80)	0.793	7.70 (6.70, 9.00)	7.90 (6.80, 8.90)	0.548	7.20 (6.10, 7.95)	6.85 (5.77, 7.60)	0.120
EOS%	2.20 (1.30, 3.30)	2.10 (1.50, 2.90)	0.717	2.40 (1.50, 3.60)	2.20 (1.60, 3.10)	0.477	1.60 (1.20, 2.60)	1.75 (1.17, 2.60)	0.676
BAS%	0.60 (0.40, 0.80)	0.50 (0.40, 0.70)	0.029	0.60 (0.40, 0.80)	0.60 (0.40, 0.70)	0.655	0.50 (0.40, 0.70)	0.40 (0.30, 0.50)	<0.001
NEU#, 10 × 9/L	3.50 (2.89, 4.45)	3.99 (3.16, 4.97)	<0.001	3.44 (2.94, 4.40)	3.88 (3.13, 4.93)	<0.001	3.58 (2.69, 4.55)	4.29 (3.33, 5.27)	<0.001
LYMPH#, 10 × 9/L	2.09 (1.66, 2.54)	2.25 (1.85, 2.72)	<0.001	2.07 (1.64, 2.52)	2.24 (1.84, 2.70)	<0.001	2.17 (1.72, 2.61)	2.26 (1.90, 2.75)	0.111
MON#, 10 × 9/L	0.49 (0.40, 0.58)	0.53 (0.43, 0.64)	<0.001	0.50 (0.42, 0.59)	0.56 (0.45, 0.66)	<0.001	0.45 (0.37, 0.53)	0.49 (0.40, 0.60)	0.060
EOS#, 10 × 9/L	0.14 (0.08, 0.22)	0.15 (0.10, 0.22)	0.047	0.14 (0.09, 0.24)	0.16 (0.11, 0.23)	0.238	0.11 (0.07, 0.17)	0.13 (0.09, 0.21)	0.089
BAS#, 10 × 9/L	0.03 (0.02, 0.05)	0.04 (0.03, 0.05)	0.202	0.04 (0.02, 0.05)	0.04 (0.03, 0.05)	0.052	0.03 (0.02, 0.04)	0.03 (0.02, 0.04)	0.420
NLR	1.68 (1.30, 2.23)	1.74 (1.36, 2.22)	0.493	1.73 (1.31, 2.27)	1.72 (1.35, 2.17)	0.723	1.56 (1.26, 2.13)	1.79 (1.37, 2.27)	0.068
LMR	4.36 (3.42, 5.26)	4.36 (3.40, 5.46)	0.747	4.12 (3.28, 5.03)	4.22 (3.31, 5.30)	0.676	4.92 (3.89, 5.68)	4.93 (3.71, 5.91)	0.965
SII	400.80 (287.89, 563.42)	427.99 (308.50, 605.89)	0.034	398.55 (286.10, 537.11)	406.13 (292.26, 557.59)	0.364	418.44 (298.80, 581.72)	456.44 (364.30, 673.78)	0.017
SIRI	0.83 (0.57, 1.18)	0.91 (0.63, 1.29)	0.012	0.89 (0.60, 1.21)	0.95 (0.64, 1.32)	0.102	0.75 (0.52, 1.14)	0.89 (0.60, 1.25)	0.034
MHR	0.42 (0.33, 0.55)	0.50 (0.40, 0.64)	<0.001	0.44 (0.35, 0.59)	0.52 (0.42, 0.67)	<0.001	0.38 (0.28, 0.46)	0.46 (0.34, 0.57)	<0.001
NHR	3.10 (2.33, 4.03)	3.77 (2.87, 4.89)	<0.001	3.17 (2.44, 4.15)	3.71 (2.89, 4.80)	<0.001	2.84 (2.05, 3.75)	4.03 (2.73, 5.06)	<0.001
TC, mmol/L	4.78 (4.13, 5.45)	4.92 (4.37, 5.57)	0.007	4.69 (4.10, 5.41)	4.92 (4.37, 5.59)	0.003	4.94 (4.33, 5.62)	4.93 (4.40, 5.51)	0.805
TG, mmol/L	1.54 (1.13, 2.24)	2.05 (1.55, 2.90)	<0.001	1.57 (1.18, 2.35)	2.12 (1.55, 3.06)	<0.001	1.45 (1.06, 2.05)	1.85 (1.55, 2.49)	<0.001
HDL-C, mmol/L	1.12 (1.00, 1.30)	1.06 (0.95, 1.20)	<0.001	1.10 (0.98, 1.24)	1.05 (0.94, 1.16)	0.001	1.25 (1.04, 1.43)	1.09 (0.97, 1.23)	<0.001
LDL-C, mmol/L	2.96 (2.37, 3.50)	3.09 (2.60, 3.62)	0.003	2.91 (2.30, 3.55)	3.05 (2.60, 3.51)	0.010	3.07 (2.47, 3.41)	3.15 (2.61, 3.68)	0.093
Serum creatinine, umol/L	59.50 (50.00, 69.00)	64.00 (52.00, 75.00)	<0.001	63.00 (56.00, 71.00)	68.00 (61.00, 78.25)	<0.001	50.00 (42.50, 56.00)	49.00 (41.75, 54.00)	0.744
Glucose, mmol/L	5.00 (4.57, 6.00)	5.00 (4.50, 5.90)	0.655	5.00 (4.50, 6.00)	4.90 (4.50, 5.62)	0.073	5.00 (4.60, 5.90)	5.40 (4.80, 6.55)	0.064

NC, neck circumference; WC, waist circumference; ESS, Epworth Sleepiness Scale; BMI, body mass index; AHI, apnea-hypopnea index; ODI, oxygen desaturation index; ARI, arousal index; REM duration, rapid eye movement sleep duration; N1 duration, stage N1 sleep duration; N2 duration, stage N2 sleep duration; N3 duration, stage N3 sleep duration; REM AHI, apnea-hypopnea index in REM sleep; NREM AHI, apnea-hypopnea index in NREM sleep; AverageSaO_2_, mean percutaneous oxygen saturation; LowestSaO_2_, minimum percutaneous oxygen saturation; T90, percentage of total sleep time with SpO_2_ < 90%; WBC, white blood cell count; RBC, red blood cell count; HGB, hemoglobin level; PLT, platelet count; NEU%, neutrophil percentage; LYMPH%, lymphocyte percentage; MON%, monocyte percentage; EOS%, eosinophil percentage; BAS%, basophil percentage; NEU#, neutrophil count; LYMPH#, lymphocyte count; MON#, monocyte count; EOS#, eosinophil count; BAS#, basophil count; NLR, neutrophil-to-lymphocyte ratio; LMR, lymphocyte-to-monocyte ratio; SII, systemic immune-inflammation index; SIRI, systemic inflammation response index; MHR, monocyte-to-HDL ratio; NHR, neutrophil-to-HDL ratio; TC, total cholesterol; TG, triglycerides; HDL-C, high-density lipoprotein cholesterol; LDL-C, low-density lipoprotein cholesterol.

**Table 3:** Logistic regression models among OSA individuals in the NHR quantiles.

Characteristic	Model 1		Model 2		Model 3	
	OR (95%CI)	*p*	OR (95%CI)	*p*	OR (95%CI)	*p*
**Male**						
NHR quantile						
Q1	1.00 (Reference)		1.00 (Reference)		1.00 (Reference)	
Q2	1.61 (0.99~2.59)	0.053	1.36 (0.82~2.26)	0.241	1.30 (0.78~2.19)	0.317
Q3	2.26 (1.40~3.65)	<0.001	1.94 (1.16~3.24)	0.011	1.75 (1.03~2.95)	0.037
Q4	2.80 (1.73~4.54)	<0.001	2.04 (1.20~3.46)	0.008	1.77 (1.03~3.06)	0.039
**Female**						
NHR quantile						
Q1	1.00 (Reference)		1.00 (Reference)		1.00 (Reference)	
Q2	1.58 (0.75~3.33)	0.230	0.97 (0.42~2.27)	0.947	0.95 (0.40~2.26)	0.912
Q3	2.78 (1.33~5.84)	0.007	1.51 (0.66~3.45)	0.328	1.42 (0.61~3.31)	0.413
Q4	4.89 (2.27~10.52)	<0.001	2.73 (1.14~6.55)	0.024	2.68 (1.10~6.51)	0.029

OR: Odds Ratio, CI: Confidence Interval; Model 1: Crude; Model 2: Adjust: age, BMI, AHI, ODI, ARI, REM AHI, NREM AHI, LowestSaO_2_; Model 3: Adjust: age, BMI, AHI, ODI, ARI, REM AHI, NREM AHI, LowestSaO_2_, CREA (umol/L), Glucose (mmol/L); BMI, body mass index; AHI, apnea-hypopnea index; ODI, oxygen desaturation index; ARI, arousal index; REM AHI, apnea-hypopnea index in REM sleep; NREM AHI, apnea-hypopnea index in NREM sleep; LowestSaO_2_, minimum percutaneous oxygen saturation; CREA, serum creatinine.

## Data Availability

No datasets were generated or analyzed in this study.

## Supplementary Materials

The following supporting information can be downloaded at https://www.mdpi.com/article/10.3390/jcm15103916/s1, Table S1: STROBE Statement checklist of items that should be included in reports of observational studies.
